# T Cell Activation and Cytokine Profile of Tuberculosis and HIV-Positive Individuals during Antituberculous Treatment and Efavirenz-Based Regimens

**DOI:** 10.1371/journal.pone.0066095

**Published:** 2013-06-19

**Authors:** Tatiana P. da Silva, Carmem B. W. Giacoia-Gripp, Carolina A. Schmaltz, Flavia M. Sant` Anna, Valeria Rolla, Mariza G. Morgado

**Affiliations:** 1 Laboratory of AIDS and Immunology Molecular, Oswaldo Cruz Institute, Fundação Oswaldo Cruz (FIOCRUZ), Rio de Janeiro, Brazil; 2 Clinical Research Laboratory on Mycobacteriosis of Instituto de Pesquisa Clínica Evandro Chagas-Fundação Oswaldo Cruz (Fiocruz), Rio de Janeiro, Brazil; Institute of Infectious Diseases and Molecular Medicine, South Africa

## Abstract

**Introduction:**

The profile of immune activation markers in tuberculosis and HIV-infected patients is already known. The impact of simultaneous infections on the immune parameters is still not fully explored.

**Methods:**

We conducted a prospective study to estimate trajectories of activated T cell subsets and the profile of anti- and pro-inflammatory cytokines in a group of HIV-TB individuals, previously naïve for HAART, recruited from a randomized clinical trial during TB treatment and first antiretroviral therapy with efavirenz. Patients were evaluated according to the immunosuppression levels at baseline as group 1 (CD4<200 cells/mm^3^) and group 2 (CD4>200 cells/mm^3^). These parameters were measured at the time of HAART initiation (started about 30 days after the onset of TB treatment) and at the follow-up visits after 30, 60, 90 and 180 days. Trajectories were estimated using least squares estimates of the coefficients of a restricted cubic spline function in time after adjusting for subject effects, bootstrapping it 500 times.

**Results:**

Increase of CD4 T cell counts and suppression of HIV viral load were observed for all patients under HAART and TB treatment. Descendent trajectories were observed for the activated CD8^+^/CD38^+^ and CD3^+^/HLA-DR^+^ T cell subsets, and for plasma concentration of gamma- interferon (IFN-γ). Except for TNF-α and IL-2 discrete variations were observed for the other cytokines. Differences in the trajectories of these parameters were observed for groups 1 and 2. Higher values of IFN-γ, IL-2, IL-6 and IL-10 were observed for group 1 from the baseline to two months after treatment initiation, whereas reduced levels of TNF-α were observed for this group between 60 and 120 days of HAART.

**Conclusion:**

Independent of the immunosuppression profile at baseline, HIV-TB patients under HAART were able to recover the CD4^+^ T cell counts, and control viral replication and immune activation parameters over time.

## Introduction

HIV-1 infection is characterized by profound immunosupression associated to inappropriate immune activation [1]. In general, after initiating highly active antiretroviral therapy (HAART), a viral suppression to undetectable levels, increase of CD4^+^ counts and a remarkable clinical improvement are observed [2]–[4]. Moreover, cellular and soluble markers of immune activation are strong predictors of HIV disease progression [5]. CD4^+^ and CD8^+^ T cell subsets expressing CD38^+^ and HLA-DR^+^ are overrepresented as a hallmark of immune activation in HIV positive patients associated or not with other pathogens [5], [6]. Despite multiple studies demonstrating increased CD38^+^ and/or HLA-DR^+^ expression on CD8^+^ T cells of HIV-TB patients [6], there are few studies showing the profile of these cellular markers along concomitant therapy for both diseases.

The pattern of cytokines produced by T lymphocytes plays a central role in susceptibility to TB [7], [8]. Activation of T helper type 1 (Th1) lymphocytes, production of interferon-γ (IFN-γ), and macrophage activation are crucial in the defense against *Mycobacterium tuberculosis* [9]. On the other hand, tumor necrosis fator-alpha (TNF-α) plays an essential role in protection but is also related to a higher risk of active disease [10].

HIV infection is associated with a profound deregulation of the immune system and alterations in the cytokine profiles. TNF also plays a pivotal role in HIV-1 pathogenesis, being found in increased levels in acute and chronic HIV-1 infections [11], [12]. T helper type 2 (Th2) cell activation and increased interleukin-4 (IL-4) production have been associated with poor clinical outcome after HIV treatment [9]. IL-6 has been considered as a biomarker for Immune Reconstitution Inflammatory Syndrome (IRIS) [13], [14] in HIV-TB patients [15].

Although the cytokines are activated as regulatory networks, the secretion of individual cytokines may provide limited information. In the present study, we conducted a prospective analysis to estimate trajectories of activated T cell subsets and the profile of anti- and pro-inflammatory cytokines produced in a group of TB-HIV individuals, previously naïve for HAART, in TB-HIV patients during treatment for both diseases and the impact of the CD4 immunosuppression levels at baseline (below or above 200 cells/mm^3^) for the trends of such parameters.

## Materials and Methods

### Patient Enrolment and Follow-up

We conducted a prospective study in a cohort of HIV positive patients with tuberculosis (TB) at the Tuberculosis Clinic of the Instituto de Pesquisa Clínica Evandro Chagas (IPEC), Fundação Oswaldo Cruz, Rio de Janeiro, Brazil, from April 2006 to July 2011. Patients were recruited from a randomized clinical trial “Randomized Clinical Trial to Assess the Efficacy and Safety of Concomitant Use of Rifampicin and Efavirenz 600×800 mg” registered at clincaltrials.gov (NCT00533390). For this trial patients were eligible if they had 18 years or more, were naïve for HAART, had TB diagnosis based on clinical signs and symptoms suggestive of tuberculosis, the radiological aspects were compatible, and other opportunistic diseases were excluded, even if specimen baciloscopy was negative. Eligibility was confirmed if the *M. tuberculosis* was identified in culture or after clinical response to treatment after two months of tuberculosis treatment (in the case of negative cultures or contamination). The exclusion criteria for the trial included: resistance to rifampicin, baseline (grade 3) hepatic enzymes elevation or CD4 counts above 350 cells/mm^3^ for patients with pulmonary tuberculosis. Therapy was adjusted to replace isoniazid or other antimycobacterial drug, if resistance was detected.

Patients were selected to the immune reconstitution study if they performed at least 3 of the scheduled visits.

HAART was initiated 30±10 days after initiation of tuberculosis therapy and included two reverse transcriptase nucleoside analogs plus efavirenz (600 or 800 mg). Follow-up visits were performed at 30, 60, 90 and 180 (30 days after the end of TB treatment) days after initiation of HAART. Blood samples were collected at baseline (15 days before HAART initiation) and at all follow-up visits to study the plasmatic immune profile. Tuberculosis and HIV therapies were prescribed according to the Brazilian Ministry of Health recommendations [16], [17].

In order to verify the association of the immune activation markers with the immune suppression profile, the patients were further distributed in two groups, according to CD4^+^ T cell counts at baseline, as follows: group 1 (<200 cells/mm^3^) and group 2 (>200 cells/mm^3^). The CD4<200/mm^3^ threshold was chosen to meet the CDC laboratory criteria for AIDS (1994).

Patients were also assessed for the onset of immune reconstitution inflammatory syndrome (IRIS) defined as a documented worsening of signs or symptoms of TB during appropriate anti-tuberculosis treatment and after the initiation of anti-retroviral (ARV) therapy, not explained by any other disease or by an adverse effect of drug therapy [18]. All IRIS patients included in the study were classified as paradoxical tuberculosis-associated IRIS according to Meintijes et al. 2008 [19], since all of them were on treatment for TB when IRIS occurred.

### Ethics Statement

This protocol was approved by the Instituto de Pesquisa Clínica Evandro Chagas Ethical Board (CAE: 0052.0.009.000-10), affiliated to the Brazilian National Ethics Council (CONEP) and all patients who accepted to participate in the study signed an informed consent form.

### CD4^+^ T Cell Counts and Viral Load Evaluation

CD4^+^ T cell counts and viral load determinations were performed according to the Brazilian guidelines as part of the routine exams for HIV positive patients. BD Multitest monoclonal antibodies specific for CD45^+^, CD3^+^, CD4^+^ and CD8^+^, respectively conjugated to PerCP, FITC, APC and PE were used to determine the absolute counts of CD4 and CD8 T cell subsets according to the manufacturer (BD Biosciences, Franklin Lakes, NJ, USA). Samples were evaluated using a FACSCalibur and analyzed with Multiset software (BD, USA). Plasma samples were obtained by centrifugation within 4 hours of blood collection and aliquots were stored at −70 C freezer until use for viral load and cytokine measurents. Quantification of the plasma viral load was determined for all HIV/−TB patients at each visit (NASBA, Organon Teknika, Boxtel,The Netherlands, and branched DNA assay, Versant HIV-1 RNA 3.0, Siemens, Tarrytown, USA), assuming the lower detection limit (LDL) of 80 copies/ml.

### Immune Activation Studies

Whole blood EDTA (50 ul) was incubated with a combination of antibodies included in the BD Simultest (BD Biosciences, USA) as follows: anti-CD8^+^ FITC/CD38^+^ PE or CD3 FITC^+^/HLA-DR^+^ PE. After 30 min incubation, the red blood cells were lysed (Becton Dickinson lysing solution), the lymphocytes were washed once with PBS pH 7,2 and fixed with (300 ul) of 1% paraformaldehyde in PBS; samples were evaluated in the FACSCalibur flow cytometer (BD Biosciences). In general, 10.000 events were collected in the lymphocyte gate by forward scatter (FSC) and side scatter (SSC) using CellQuest™ Software (BD Biosciences). CD3 or CD8 T cell populations were determined in the lymphocyte population based on the forward scatter *vs* fluorescence dot plot. HLA-DR and CD38 percentages were defined in CD3 and CD8^high^ T cell populations, respectively.

### Immunoassays for Cytokines

Plasma level of cytokines (IL-2, IL-4, IL-6, IL-10, TNF-α, IFN-γ) were measured using the Cytometric Beads Array kit (CBA, BD Biosciences, San Diego, CA, USA) using a FACSCalibur cytometer. The reagents and samples were prepared according to the manufacturer’s instructions.

### Statistical Analysis

Viral load and immunological parameters (CD4 T cell subset, cytokine levels and the profile of activated T cells) were assessed for the HIV-TB patients along the follow-up treatment. The patients were further distributed in two groups according to CD4 T cell counts at study entrance: in <200 cells/mm^3^ (group 1) and >200 cells/mm^3^ (group 2). Mann whitney test, as well as Fisher’s exact and Chi-square tests were employed for this study and a P value <0.05 was considered significant.

Trajectories of markers were estimated as follow: least squares estimates of the coefficients of a restricted cubic spline function in time after adjusting for subject effects through the use of subject dummy variables. Then the fit was bootstrapped 500 times, by treating time and subject as random variables. Samples are taken jointly from the time, subject, and response vectors to obtain unconditional distributions.

The bootstrap sampling was based on samples with replacement from subjects rather than from individual data points. In other words, either none or all of a given subject’s data will appear in a bootstrap sample. This cluster sampling takes into account any correlation structure that might exist within subjects, so that confidence limits are corrected for within-subject correlation. Assuming that ordinary least squares estimates, which ignore the correlation structure, are consistent (which is almost always true) and efficient (which would not be true for certain correlation structures or for datasets in which the number of observation times vary greatly from subject to subject), the resulting analysis will be a robust, efficient repeated measures analysis for the one-sample problem. The method of Tibshirani was used to obtain simultaneous confidence sets for the set of coefficients of the spline function as well as the average intercept parameter (over subjects).

Statistical analysis was done using the SPSS Software version 17.0 and for the graphics R-project 2.15.3 software with package Hmisc.

## Results

In total, seventy HIV-TB patients agreed to participate in the study. Sixteen patients were thereafter excluded due to non-adherence to the HIV treatment (n = 7) or for attending less than 3 follow-up visits along treatment (n = 9). Demographic, clinical and laboratory data (viral load and CD4^+^ T cell counts) of the remaining 54 HIV-TB patients at baseline are shown in Table 1. In this group we observed a predominance of males and a similar proportion of pulmonary and disseminated tuberculosis. Moreover, the median CD4^+^ T cell count and viral load were 112 cells/mm^3^ and 5.02 log_10_copies/ml, respectively, with predominance of heavily immunosuppressed patients (61%). In spite of that, few cases of IRIS were observed in our cohort (11%). Moreover, the HIV-TB patients included in this cohort were treated with two distinct doses of efavirenz (600 mg or 800 mg/day). As no differences were observed for the virological and immunological parameters here analyzed, they were considered as a single group (data not shown).

**Table 1:** Baseline demographic clinical laboratorial and therapeutical characteristics in HIV-TB patients.

Characteristic	N = 54
Age (years), median (IQR)	37.5 (32–43)
Gender, n (%)	
Male	42 (77.8)
Female	12 (22.2)
Site of tuberculosis, n (%)	
Pulmonary	24 (44.4)
Disseminated	26 (48.1)
Extrapulmonary	4 (7.4)
Diagnosis of tuberculosis, n (%)	
Positive culture	35 (65)
Histopatological criteria	5 (9)
Clinical criteria	14 (26)
Efavirenz dose, n (%)	
600 mg	27 (50)
800 mg	27 (50)
IRIS, n (%)	
Yes	6 (11)
No	48 (89)
Baseline CD4, median (IQR)	112 (46.5–236.2)
<200 cells/mm^3^	55 (33.5–104)
>200 cells/mm^3^	262 (231–389)
Baseline CD4, n (%)	
<200 cells/mm^3^	33 (61)
>200 cells/mm^3^	21 (39)
Baseline HIV-1 viral load (log_10_copies/ml),median (IQR)	5.02 (4.47–5.56)

N = number of cases.

**Figure 1:**
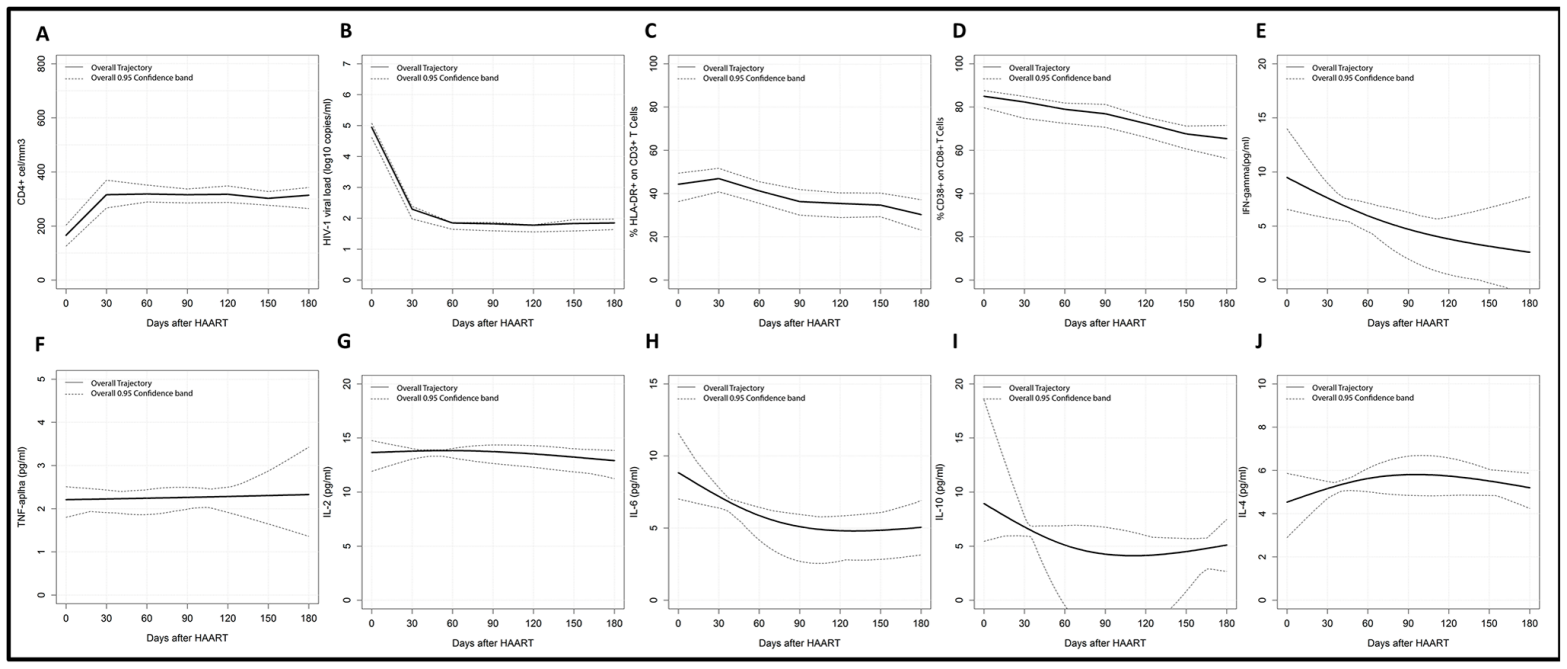
Graphic representation of the overall trajectories of immunological and virological biomarkers in HIV-TB co-infected patients along TB treatment and HAART. (A) CD4, (B) HIV-1 viral load, (C) % of CD3+ T cells expressing HLA-DR+, (D) % of CD8+ T cells expressing CD38+, (E) IFN-γ, (F) TNF-α, (G) IL-2, (H) IL-6, (I) IL-10 and (J) IL-4.

Changes in the immune activation markers, CD4^+^ T cells counts, cytokine levels and viral load along HAART and TB treatment are shown in the Table 2. The immune activation was defined by the frequency of CD3^+^ and CD8^+^ T cell subsets expressing HLA-DR^+^ and CD38^+^ molecules, respectively. The trajectories obtained for these parameters along the follow-up are presented in the Figure 1 (A–D). Significant increase in the CD4 counts and decrease of viral load were observed up to 30 days of HAART, and later periods did not show an increase or decrease trend. For CD3^+^/HLA-DR^+^, descendent trajectory was observed after 30 days of HAART, followed by a continuous descent overtime curve. Reduction of the CD38^+^/CD8^+^ T cell subsets were observed over time. It is interesting to note that extremely high activation levels of T cells, especially of CD8^+^ T cells, were observed for HIV-TB patients, at the beginning of tuberculosis treatment and HAART.

**Table 2:** Changes in the virological and immunological profiles, including immune activated T cell subsets and cytokines, in HIV-TB patients during TB treatment and HAART.

Variable	Follow-up visits				
	Baseline	30 days	60 days	90 days	180 days
CD4+ T cell	112 (46–236)	245 (151–452)	264 (151–403)	287 (144–478)	291 (167–439)
HIV-1 Viral load	5.0 (4.4–5.5)	2.2 (1.8–2.7)	1.7 (1.7–1.8)	1.7 (1.7–1.7)	1.7 (1.7–1.7)
HLA-DR^+^/CD3^+^	39.6 (28–60)	45 (29–62)	42 (28–57)	29 (20–59)	24 (16–38)
CD38^+^/CD8^+^	90 (79–97)	86 (72–92)	85 (75–94)	77 (62–89)	70 (50–80)
IFN-γ (pg/ml)	1.9 (0.0–10.1)	2.0 (0.0–11.8)	1.1 (0.0–7.7)	0.25 (0.0–5.4)	0.01 (0.0–5.3)
TNF-α (pg/ml)	2.2 (0.0–3.1)	2.3 (0.0–3.2)	2.2 (0.0–3.1)	2.2 (0.0–2.9)	2.4 (0.5–3.4)
IL-2 (pg/ml)	10.8 (8.0–12.6)	10.7 (8.3–14.8)	10.8 (8.6–14.3)	10.7 (8.5–12.2)	10.8 (9.0–13.5)
IL-6 (pg/ml)	4.3 (1.5–9.4)	5.2 (2.6–12.7)	3.6 (1.7–6.5)	2.5 (0.8–5.0)	3 (1.3–5.0)
IL-10 (pg/ml)	2.6 (0.3–6.0)	2.9 (0.5–7.0)	2.4 (0.8–4.9)	2.0 (0.4–3.7)	2.5 (1.4–3.9)
IL-4 (pg/ml)	4.3 (1.4–8.5)	5.1 (2.1–9.0)	5.9 (1.6–8.0)	4.8 (0.7–8.7)	3.3 (0.3–8.9)

Values are expressed as median (IQR); CD4^+^ T cell count (cells/mm^3^); HIV-1 viral load (log_10_copies/ml); HLA-DR^+^/CD3^+^: %HLA-DR^+^ molecule on CD3^+^ T cells; CD38^+^/CD8^+^:% CD38^+^ molecule on CD8^+^ T cells; IFN-γ: Interferon-gamma; TNF-α: Tumor necrosis factor-alpha; IL: Interleukin.

A panel of pro-inflammatory and regulatory cytokines (Table 2) was tested in plasma samples collected along the study in order to try to associate immune activation and cytokine profiles. Figure 1 (E–J) shows the analyses of the trajectories of these markers along the treatment for tuberculosis and aids. A decreasing trend of plasma concentration of gamma- interferon (IFN-γ) was observed overtime. Except for TNF-α and IL-2 that did not change along treatment, for the other cytokines discrete variations were observed.

Demographic, clinical and laboratory data of the groups 1 and 2, distributed according to the CD4^+^ T cell counts at baseline, are shown in Table 3. Except for the CD4^+^ T cell counts, viral load and IFN-γ levels, no statistically significant differences was observed for the other parameters between the two groups.

**Table 3:** Baseline demographic characteristics of HIV-TB patients distributed according to the T CD4 count.

Characteristic	CD4<200 cell/mm^3^ (N = 33)	CD4>200 cell/mm^3^ (N = 21)	P value*
Age (years), median (IQR)	38 (32–43)	35 (31–40)	0.424^a^
Gender, n (%)			
Male	26 (79)	16 (76)	0.539^b^
Female	7 (21)	5 (24)	
Site of tuberculosis, n (%)			
Pulmonary	14 (43)	10 (48)	0.784^c^
Disseminated	17 (51)	9 (43)	
Extrapulmonary	2 (6)	2 (9)	
Diagnosis of tuberculosis, *n* (%)			
Positive culture	22 (67)	13 (62)	0.467^c^
Histopatological criteria	4 (12)	1 (5)	
Clinical criteria	7 (21)	7 (33)	
IRIS cases, n (%)			
Yes	5 (15)	1 (4)	0.236^b^
No	28 (85)	20 (96)	
Efavirenz dose, n (%)			
EFV 600 mg	16 (48)	11 (52)	0.456^b^
EFV 800 mg	17 (52)	10 (48)	
Baseline CD4(cells/mm^3^), median (IQR)	55 (33–104)	262 (231–389)	0.000^a^
Baseline HIV-1 viral load (log_10_copies/ml), median (IQR)	5.2 (4.8–5.7)	4.8 (4.1–5.2)	0.006^a^
HLA-DR^+^/CD3^+^ median (IQR)	39.6 (29.7–62.8)	38.5 (21.1–55)	0.333^a^
CD38^+^/CD8^+^ median (IQR)	89.3 (79–97.8)	87.7 (70.5–90.4)	0.090^a^
IFN-γ median (IQR)	2.4 (0–16)	0 (0–5.2)	0.012^a^
TNF-α median (IQR)	1.4 (0–3.1)	2.5 (1.9–3)	0.224^a^
IL-2 median (IQR)	10.9 (7.9–17.9)	10.6 (10–11)	0.747^a^
IL-6 median (IQR)	5.7 (0–12.3)	3.8 (2.5–5.3)	0.294^a^
IL-10 median (IQR)	3.8 (0–11.7)	2.2 (1.8–4.9)	0.657^a^
IL-4 median (IQR)	4.3 (2.3–7.8)	6.1 (0.2–9.2)	0.779^a^

N = number of cases;

Mann Whitney test^a^, Fisher’s exact test^b^ and Chi-square test^c^;

IRIS-Immune reconstitution inflammatory syndrome;

EFV = efavirenz.

**Figure 2:**
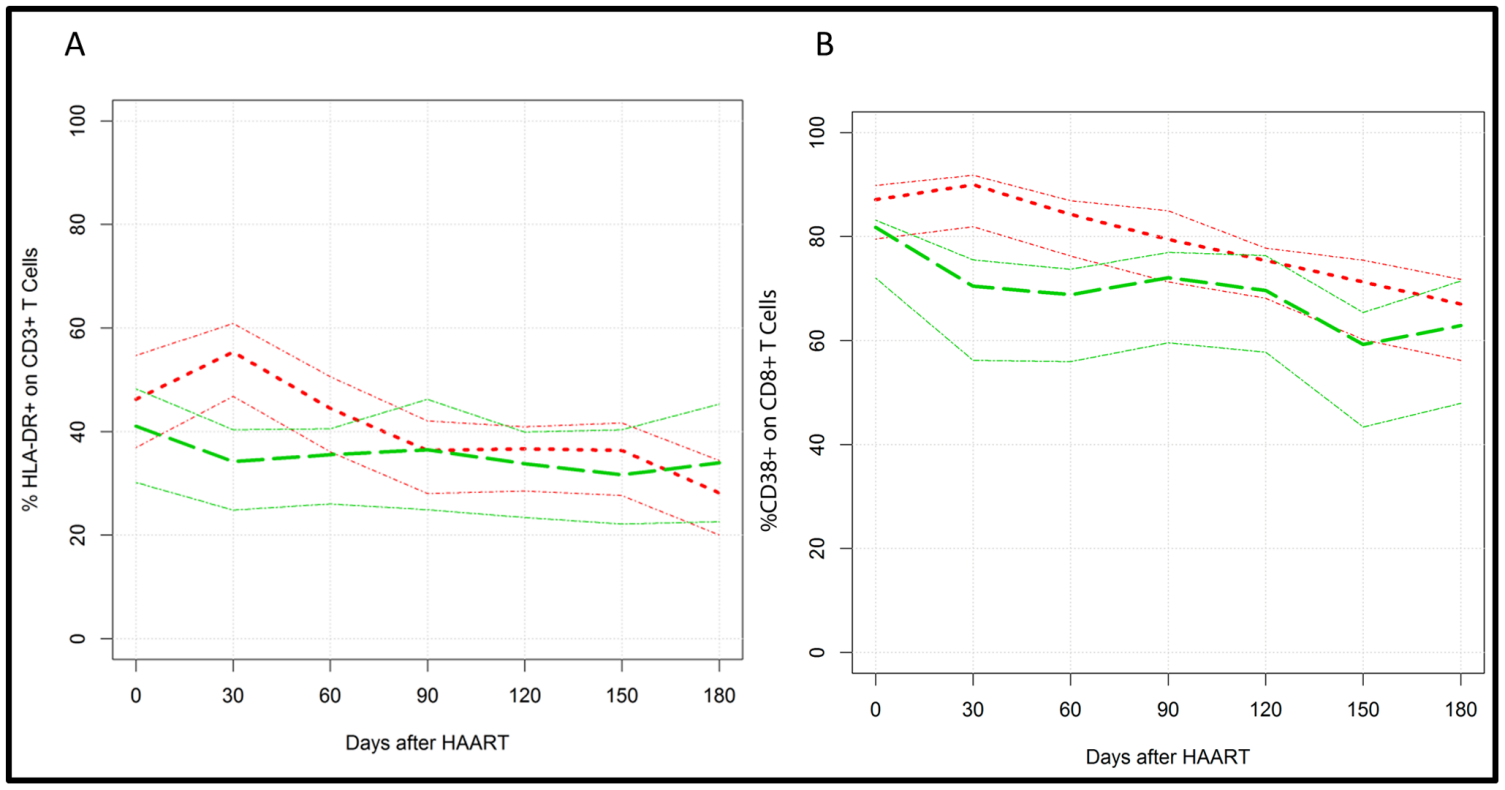
Graphic representation of the trajectories of the immune activation T cell subsets in HIV-TB co-infected patients along TB treatment and HAART, distributed according to CD4+ T cell counts (<200 cell/mm^3^ and >200 cell/mm^3^). (A) % of CD3+ T cells expressing HLA-DR+ and (B) % of CD8+ T cells expressing CD38+. Overall trajectories are represented for red dotted lines for <200 cell/mm^3^ group and the green dotted line for >200 cell/mm^3^ group. The thin lines represent the overall 0.95 confidence bars.

**Figure 3:**
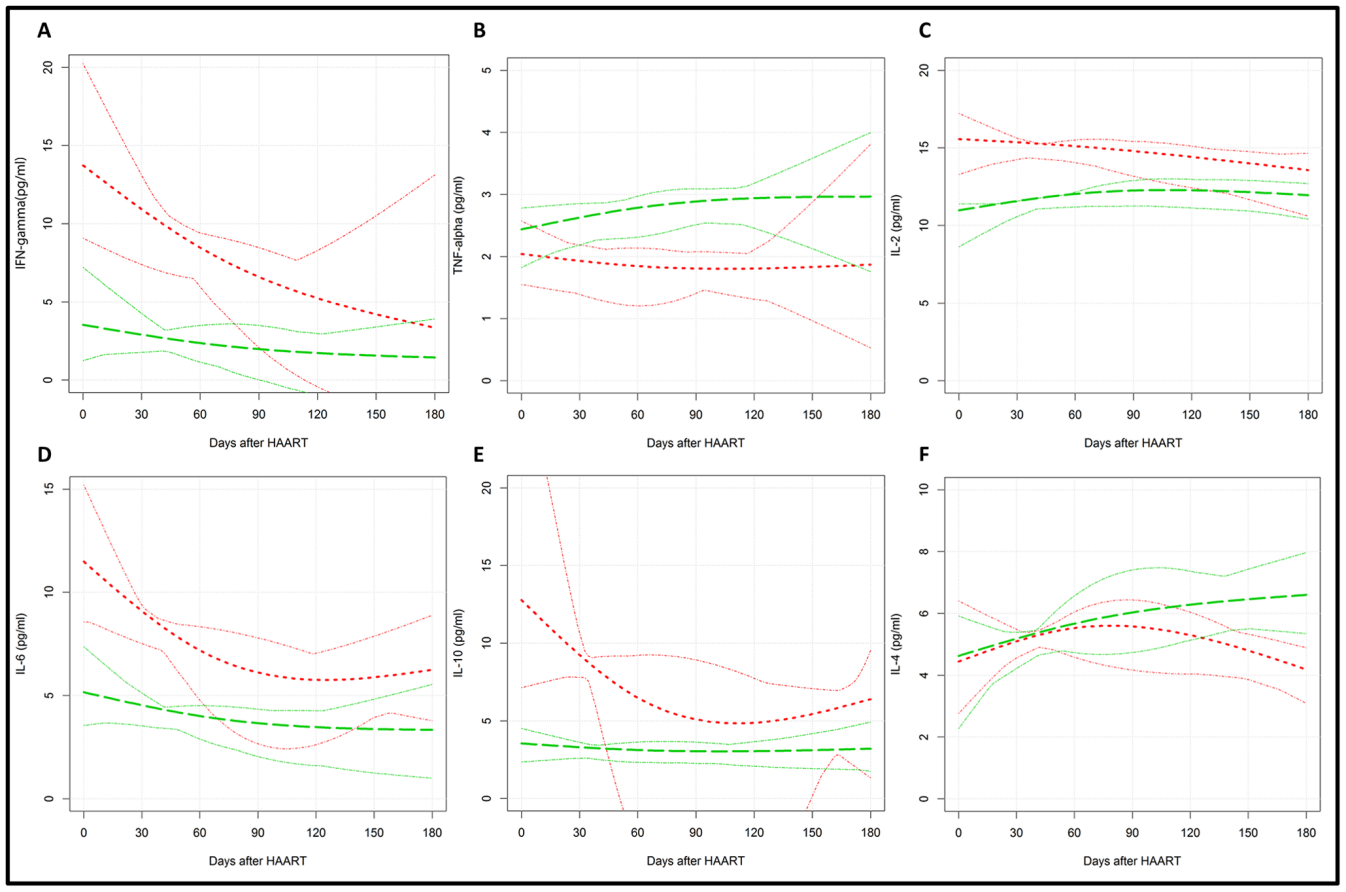
Graphic representation of the trajectories of pro- and anti-inflammatory cytokines in HIV-TB co-infected patients along TB treatment and HAART, distributed according to CD4^+^ T cell counts (<200 cell/mm^3^ and >200 cell/mm^3^). (A) IFN-γ, (B) TNF-α, (C) IL-2, (D) IL-6, (E) IL-10 and (F) IL-4. The plasma samples were tested by Cytometric Bead-Array (CBA) Overall trajectories are represented for red dotted lines for <200 cell/mm^3^ group and the green dotted line for >200 cell/mm^3^ group. The thin lines represent the overall 0.95 confidence bars.

The trajectories curves of the immune activation T cell profiles and pro- and anti-inflammatory cytokine levels were compared between the groups 1 and 2 along HAART and tuberculosis treatment. In fact, CD3^+^/HLA-DR^+^ and CD38^+^/CD8^+^ T cell subsets were similar between the two groups at several time points, with exception for the 30 days visit for CD3^+^/HLA-DR^+^, and 30 and 60 days visit for CD38^+^/CD8^+^ T cell subset, despite viral load control (Fig. 2A and B). Higher values of IFN-γ, IL-2, IL-6 proinflammatory cytokines and IL-10 were observed for the heavily immunosuppressed group (group 1) from the baseline to two months after treatment initiation, whereas reduced levels of TNF-α were observed for this group between 60 and 120 days of HAART (Fig. 3 A–F).

## Discussion

The role of the chronic immune activation and inflammation in HIV pathogenesis, although paradoxical, has become increasingly evident [1]. Concomitant infections can exert an impact on viral replication by contributing to an increase in the T lymphocyte activation already seen in HIV patients [20]. So, in the present study, we evaluated the profile of T cell activation and anti and pro-inflammatory cytokines production in HIV-TB patients along treatment for tuberculosis and HAART.

In general, significant recovery of CD4^+^ T cell counts and control of viral replication were obtained along the follow-up treatments. Moreover, decrease of immune activated T cell subsets CD8^+^/CD38^+^ and CD3^+^/HLA-DR^+^ were observed after HAART initiation, resulting in a satisfactory recovery of the cellular immune response, in accordance with others studies [11], [20]. Although the more accurate marker used to assess CD8^+^ T cell activation is the co-expression of CD38 and HLA DR, based on our previous experience we used the detection of CD38 in CD8^+^ as a surrogate of this marker [21]. Moreover, no investigation was performed to T CD4^+^ cells, since recently different groups have shown that CD38 in CD4 does not seem to add information on the activation status of lymphocytes [22], [23]. Thus to achieve a general evaluation of T lymphocytes activation, CD3^+^/HLA-DR^+^ cells were analyzed. This cellular profile is altered during bacterial disease/sepsis and differs in patients presenting pulmonary multidrug-resistant and drug-sensitive tuberculosis [24], [25]. Similar results were observed by Lancioni *et al* in patients starting HIV-TB treatment with CD4^+^ T cells >350 cells/mm^3^ [26], whereas in our cohort patients were included with CD4^+^ T cell counts from 7 to 660 cells/mm^3^, with 61% of heavily immunosuppressed HIV-TB patients. A previous study from our group also demonstrated a similar scenario for HIV-Leprosy cases, for which high percentages of CD38^+^/CD8^+^ T cell were observed, according to HIV viral load levels, with reduction of cellular activation being achieved after HAART control of viral replication [27].

Cytokines are important immunomodulating agents of the immune system. IFN-γ and TNF-α have key roles in HIV-TB infection and are associated with inflammatory granuloma organization, immune protection against intracellular mycobacteria and cell mediated immunity [28], [29], [30]. Decrease in plasmatic IFN-γ levels were observed in our cohort all along the treatment, however TNF-α and IL-2 levels did not change during the follow-up, in spite of CD4^+^ T cell recovery and viral control. Lower IL-2 and TNF-α plasmatic levels were observed in our study than those reported by others including HIV-TB co-infected patients [9], [15]. These differences can possibly be due to the employed methodological approaches for cytokine measurement, type of biological material (plasma *vs* stimulated cell culture supernatants) or levels of immunosuppression of the HIV-TB patients. Moreover, Bal *et al*., evaluating *in vitro* stimulated T cell response to mitogens, found lower IFN-γ, TNF-α and IL-2 cytokine levels when comparing TB positive patients, HIV positive or HIV-TB co-infected ones, suggesting that HIV could be responsible for negatively regulating both cytokines [31].

IL-6 plays a role in the development of the inflammatory response during immune restoration and can act as a marker for persistent immune activation [13]. Moreover, this cytokine is also considered as a biomarker for IRIS in HIV-TB patients under HAART [13]. High levels of IL-6 were observed in our cohort at baseline, followed by a significant reduction 30 days after HAART and afterwards. However, no differences were observed when IRIS and non-IRIS patients were analyzed, indicating that by itself IL-6 levels was not a reliable marker for IRIS in our study group.

We further hypothesized that the immunosuppression level of HIV-TB patients at baseline could influence the immune activation profile and the cytokine production in response to the tuberculosis treatment and HAART. For that reason, we evaluated those parameters in HIV-TB patients along both treatments with emphasis in their immunosuppression status according to the CD4^+^ T cells counts as <200 (group 1) and >200 cells/mm^3^ (group 2).

Although no differences in the T cell activation markers were observed at baseline for the patients included in groups 1 and 2, higher CD3^+^/HLA-DR^+^ and CD38^+^/CD8^+^ T cell activation profiles were observed for group 1 after HAART initiation, followed by a slight reduction of these immune activation markers along the HIV-TB treatment for group 1. This finding suggests that patients with severe immunosuppression under HAART initially have a cellular activation more intense than those included in group 2, independent of viral control. If the presence of *Mycobacterium tuberculosis* antigens is also playing a role to support this immune activation is still an open question. Two recent studies from a prospective HIV-tuberculosis cohort treated for tuberculosis, conducted in Uganda showed that TB therapy strongly reduced the expression of CD38 and/or HLA-DR expression on CD8 T while for CD4 cells the reduction in the expression of these markers was less pronounced. Similar results were observed for HIV-tuberculosis patients receiving antiretroviral therapy concurrent with tuberculosis treatment, highlighting the relationship among *M. tuberculosis*, immune activation and circulating T cell CD8^+^/CD38^+^ cells [6], [26].

In fact, in a previous study from our group we observed that *Leishmania antigens* can increase the degree of immune activation in individuals coinfected with HIV [32]. In addition, those with American visceral leishmaniasis, with profound immune suppression before HAART, can present low CD4^+^ T cell counts and higher proportion of activated T lymphocytes even when HIV viral load is suppressed under HAART.

In general differences on the IFN-γ, IL-2, IL-6 and IL-10 levels between the two groups were more evident at baseline and just after the beginning of HAART. Although IL-10 and IL-4 are anti-inflammatory cytokines related to immune suppression [33], different profiles were observed between group 1 and 2. Whereas we did not observe significant differences between both groups either at baseline or along the HIV-TB treatments for IL-4, lower levels of IL-10, in HIV-TB patients with CD4>200 were observed at baseline compared with those with CD4<200, in agreement Subramanyam *et al.* [9].

Distinct profiles of IFN-γ and TNF-α pro-inflammatory cytokines were observed according to the immunosuppression status of the HIV-TB patients included in our study. Indeed, higher IFN-γ levels were observed for the heavily immunosuppressed group from baseline to 60 days after HAART, whereas no variation was observed for the TNF-α levels overtime. Higher levels of plasma IFN-γ were also observed by Subramanyam *et al.* [9] in patients with CD4<200 cells/mm^3^.

Some limitations of the current study should be noted mainly in relation to the low incidence of paradoxical tuberculosis-associated IRIS cases. Indeed, a recent study from Cambodia comparing early versus later start of HAART in adult HIV-TB patients showed a high frequency of IRIS cases, with differences according to the time of HAART introduction, showing significant increase of IRIS in the earlier-HAART group [34], [35]. It is of note the low CD4^+^ T cell median counts observed for that study group (25 cells/mm^3^ IQR 10–55). The low incidence of paradoxical tuberculosis-associated IRIS observed in our study group was probably due to the high CD4^+^ counts at baseline (112 cells/mm^3^ IQR 46–236) and the time point to introduce HAART which was longer than in the Cambodia study. Moreover, genetic background of the two populations could also be implicated in the risk of developing IRIS. No deaths were observed among IRIS patients in the present study. The low number of cases could explain this finding. Previous study of our group and other authors had also shown a low incidence of IRIS deaths [36], [37].

In conclusion, our results indicate that independent of the immunosuppression profile, HIV-TB patients under tuberculosis treatment and HAART are able to recover the CD4^+^ T cell counts and control of viral replication and immune activation over time. No specific pro and anti-inflammatory cytokine patterns could be observed in our study, although higher IFN-γ levels have been observed in the heavily immunosuppressed group associated to the increase of the CD4^+^ T cell counts and immune activated T cell subsets early upon HAART initiation.
